# A pseudouridylation switch in rRNA is implicated in ribosome function during the life cycle of *Trypanosoma brucei*

**DOI:** 10.1038/srep25296

**Published:** 2016-05-04

**Authors:** Vaibhav Chikne, Tirza Doniger, K. Shanmugha Rajan, Osnat Bartok, Dror Eliaz, Smadar Cohen-Chalamish, Christian Tschudi, Ron Unger, Yaser Hashem, Sebastian Kadener, Shulamit Michaeli

**Affiliations:** 1 The Mina and Everard Goodman Faculty of Life Sciences and Advanced Materials and Nanotechnology Institute, Bar-Ilan University, Ramat-Gan 52900 Israel; 2 Department of Biological Chemistry, The Alexander Silberman Inst. of Life Sciences, The Hebrew University of Jerusalem, Edmond J. Safra Campus, Jerusalem 91904, Israel; 3 Department of Epidemiology and Microbial Diseases, Yale School of Public Health, New Haven, CT 06536, USA; 4 Architecture et Réactivité de l’ARN UPR9002, Université de Strasbourg, 67084 Strasbourg, France

## Abstract

The protozoan parasite *Trypanosoma brucei,* which causes devastating diseases in humans and animals in sub-Saharan Africa, undergoes a complex life cycle between the mammalian host and the blood-feeding tsetse fly vector. However, little is known about how the parasite performs most molecular functions in such different environments. Here, we provide evidence for the intriguing possibility that pseudouridylation of rRNA plays an important role in the capacity of the parasite to transit between the insect midgut and the mammalian bloodstream. Briefly, we mapped pseudouridines (Ψ) on rRNA by Ψ-seq in procyclic form (PCF) and bloodstream form (BSF) trypanosomes. We detected 68 Ψs on rRNA, which are guided by H/ACA small nucleolar RNAs (snoRNA). The small RNome of both life cycle stages was determined by HiSeq and 83 H/ACAs were identified. We observed an elevation of 21 Ψs modifications in BSF as a result of increased levels of the guiding snoRNAs. Overexpression of snoRNAs guiding modification on H69 provided a slight growth advantage to PCF parasites at 30 °C. Interestingly, these modifications are predicted to significantly alter the secondary structure of the large subunit (LSU) rRNA suggesting that hypermodified positions may contribute to the adaption of ribosome function during cycling between the two hosts.

Pseudouridine (Ψ) is the most abundant RNA modification. In yeast 46 Ψs exist on rRNA whereas in human 91 positions are pseudouridylated^1^. This modification not only was found in rRNA, tRNA and snRNA molecules, but on mRNAs, as well as in small nucleolar RNAs (snoRNA) in both yeast and humans^2^,^3^,^4^,^5^.

Pseudouridines increase the potential for formation of an extra hydrogen bond compared to uridine and contribute to structural stability and stacking interactions of the RNA^1^. The isomerization of uridine is mediated by pseudouridine synthase. This enzyme is either bound to the H/ACA snoRNAs, which guide the modification by non-continuous 10–12 nt complementarity to their target site^6^,^7^, or by soluble enzymes^8^. H/ACA snoRNAs are present in eukaryotes, but not in bacteria, and mostly guide modifications on rRNA^9^. In yeast, the pseudouridine synthase CBF5 of the H/ACA snoRNPs is essential for growth^10^ and mutations of this enzyme in humans causes diseases including cancer^11^. Although individual Ψs may have a minor effect on the function of the RNA, a combination of Ψs present in certain domains affect ribosome processing and translation fidelity^12^,^13^. Finally, recent studies showed that in yeast, a Ψ on U2 snRNA is induced by nutrient deprivation or heat shock^14^ and that hundreds of Ψs are induced on mRNA during heat shock or nutrient depletion^3^,^5^.

*Trypanosoma brucei* (*T. brucei*) cycles between two hosts requiring major adaptation to changes in temperature and nutrients^15^. However, little is known about the molecular mechanism(s) mediating the adaptation to these very different environments. In particular, there is no information how trypanosome ribosomes can be fully functional at such dissimilar temperatures. Trypanosomes possess a rich repertoire of snoRNAs^16^,^17^. H/ACA are unique as they are composed of only a single hairpin compared to double hairpin structure found in most eukaryotes^18^,^19^. A recent study identified 63 H/ACA RNAs and 79 C/D snoRNAs, which guide 2′-*O*-methylation on rRNA^16^. It was suggested that the high hypermodification in this organism as compared to yeast with a similar genome size that has only 46 Ψs, may result from the need to preserve ribosome function during cycling between the two hosts^20^,^21^. Silencing of CBF5 (H/ACA pathway) and NOP1 (C/D pathway) in *T. brucei* demonstrated that both snoRNA families are essential and their depletion affected the complex rRNA processing in these parasites^20^,^21^.

In this study, we performed Ψ-seq on rRNA of the two life cycle stages, namely procyclic form (PCF) and bloodstream form (BSF) of *T. brucei*. Small RNome analysis indicated that H/ACA snoRNAs are developmentally regulated. We detected 83 H/ACA RNAs expressed in the two stages. Moreover, we mapped 68 Ψs to the rRNA and 21 of them were hypermodified in BSF. The snoRNAs guiding these hypermodified Ψs are up-regulated in BSF due to the elevated level of their precursors. Interestingly, four domains of the large subunit (LSU) rRNA were shown to be hypermodified; four positions on H69, four on H89, and one each on H90 and H92 located in the peptidyl-transferase center (PTC), possibly assisting in the function of the ribosome in BSF trypanosomes. Indeed, overexpression of snoRNAs which guide the Ψs on H69 improved the growth of PCF at an elevated temperature compared to their normal growth temperature, suggesting that the hyper-pseudouridylation contributes to the adaptation of the parasite while cycling between the two hosts and experiencing a temperature change of 10 °C. This is the first study suggesting that rRNA pseudouridylation is not static but it is developmentally regulated.

## Results

### Twenty-one Ψs are increased in the parasite bloodstream form compared to the procyclic form

**Figure 1:**
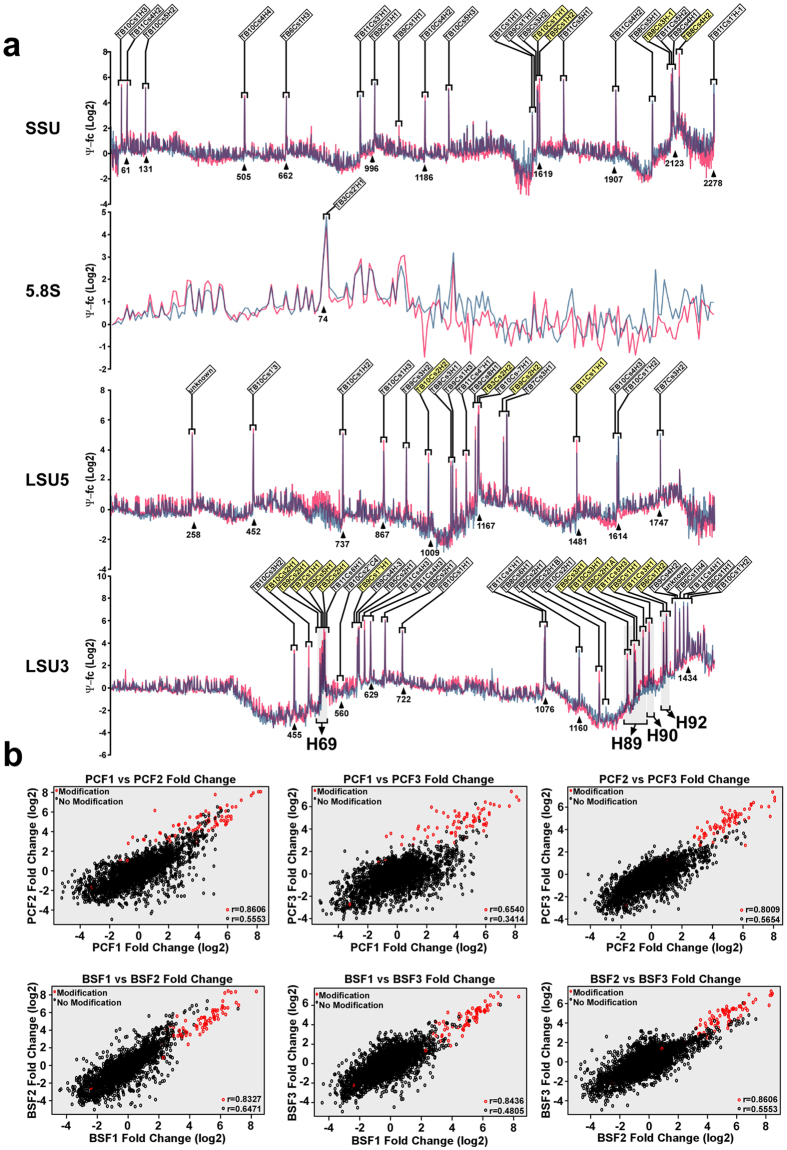
Ψ -seq detects sites that were predicted by their guiding snoRNA. **(a)** Total RNA from BSF and PCF was treated with CMC, fragmented and used to prepare a library for RNA-seq as described in “Methods”. The reads were mapped and the Ψ- fold change (Ψ-fc) values (y-axis) were determined as described in “Methods”. The Ψ-fc is the log2 transformed. Ψ-ratio of the treated samples (+CMC) divided by the Ψ-ratio in the non-treated samples (−CMC). The Ψ-fold change was computed for the PCF and BSF (mean of the three biological replicates for each condition). The values (at each position on the SSU (2280 nt), LSU5′ (1920 nt) and LSU3′ (1496 nt) and 5.8S (209 nt) rRNA) are given and plotted; BSF is pink and PCF in light blue. The black arrows indicate the snoRNA guiding the modification. The hypermodified positions are highlighted in yellow and the average for each position was plotted. (**b**) Scatterplots of the pairwise comparisons of Ψ-fc across independent replicates demonstrate the reproducibility of Ψ**-**seq for known pseudouridiylated sites (in red) versus non-modified sites (in black) both in procyclic and bloodstream forms. Pearson’s correlation coefficient is indicated on each scatterplot for modified and non-modified sites.

**Figure 2:**
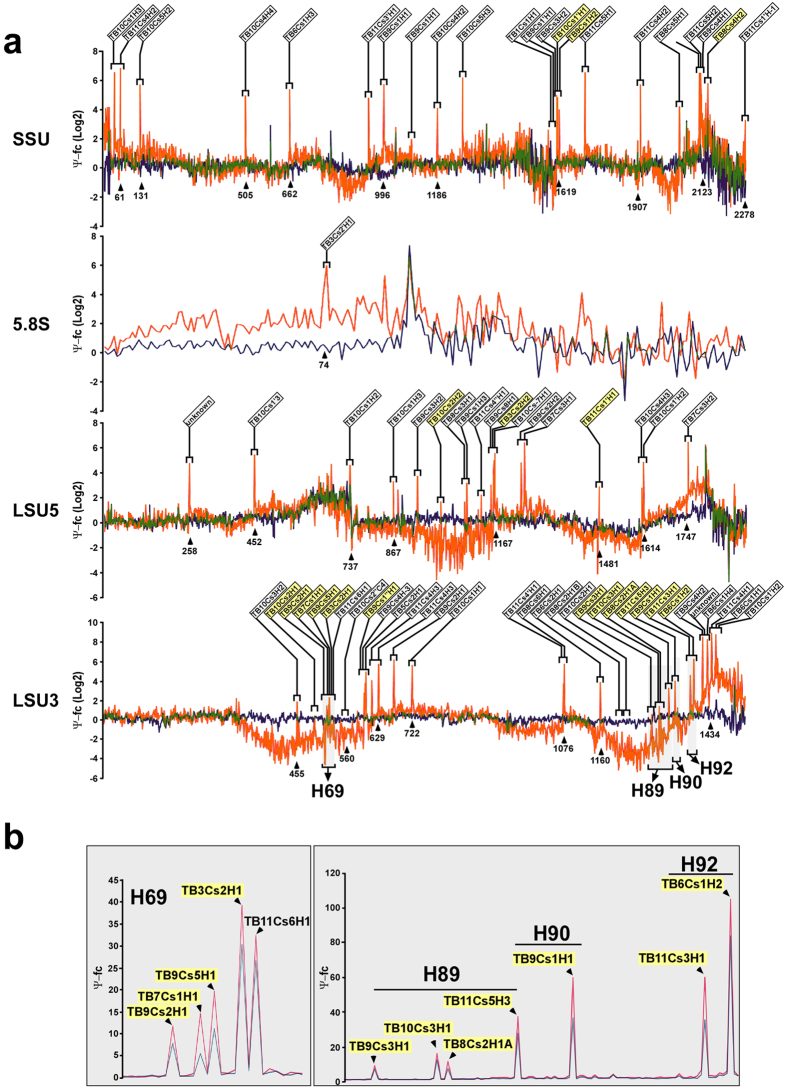
(**a**) The silencing of *CBF5* eliminates the Ψs on rRNA. Cells carrying the silencing construct for *CBF5* were silenced for 2.5 days, RNA was treated with CMC and the reads obtained from uninduced cells (-Tet) treated with CMC compared to reads obtained from silenced cells (+Tet) treated with CMC. The values (at each position on the SSU, LSU5′ and LSU3′ and 5.8S rRNA) are given and plotted; RNA obtained from un-induced cells carrying the *CBF5* silencing construct and treated with CMC (orange) and RNA from the *CBF5* silenced cells treated with CMC (blue). The experiment was repeated, and the average fold-change for each position was plotted. Note that the line (blue) representing *CBF5* silenced reads is virtually flat. **(b)** The hypermodified sites on BSF. Presentation of the Ψ- fold change (Ψ-fc) of a subset of hypermodified sites is depicted in an arithmetic scale; BSF values are in pink and PCF in light blue. The snoRNAs guiding the hypermodification are indicated with a yellow flag.

The prediction of Ψs on *T. brucei* and *Leishmania* rRNA is based mostly on the presence of snoRNAs, which are known to guide this modification. A large number of these modifications are trypanosome-specific. To verify that the predicted modifications exist, we mapped the Ψ across the rRNA based upon CMC (N-cyclohexyl-N’-β-(4-methylmorpholinium) ethylcarbodiimide p-tosylate) modification followed by alkaline treatment. Under these conditions, the addition of CMC in the place of the Ψ results in inefficient reverse transcription during the library preparation process, with the reverse transcription product terminating one nucleotide before the modified base^22^. We prepared RNA-seq libraries from total RNA from PCF and BSF parasites with and without CMC treatment. In order to identify all Ψs, we utilized pair-end sequencing. To locate the Ψs on rRNA, we used a recently published analysis pipeline^2^ which determines the ratio of the number of reads supporting reverse transcriptase termination to the number of reads overlapping it (known as the Ψ-ratio). The Ψ-fold change (Ψ-fc) is the log2-transformed Ψ-ratio of the treated samples (+CMC) divided by the Ψ-ratio in the non-treated samples (−CMC). Indeed and as expected, the Ψ-ratio identified a single strong peak one base downstream of the modified site. The average Ψ-fc (3 replicates) on SSU and LSU in the two life stages are presented in Fig. 1A. Comparing the Ψ-fc across replicates showed that there was a moderate positive correlation between the samples for non-modified sites, and a high correlation for the known modified sites (averaged Pearson correlation coefficient: for modified sites r = 0.81; for non-modified sites r = 0.47; p-value < 2.2e–16 for all pairwise comparisons). The scatterplots are presented in Fig. 1B. To determine if these Ψs are all directed by snoRNAs, the Ψ-seq was performed on cells depleted of *CBF5* by RNAi^21^. Interestingly, all the peaks seen in the control were significantly diminished in the *CBF5*-silenced cells (Fig. 2a), indicating that like in other eukaryotes, the H/ACA snoRNAs direct the Ψs on rRNA. We identified a total of 76 Ψs in the two life stages. 69 of them were predicted to exist based on snoRNAs described in an earlier study^16^, and 62 of those are supported by the Ψ mapping. Interestingly, we found 14 additional modifications by Ψ-seq. Eight sites were on uridines adjacent to previously described modifications. Earlier studies also noticed that the reverse transcriptase stop may arise from the “stuttering” of the reverse transcription leading to this artifact^22^. Thus, these eight stops are most probably due to the adjacent sites suggested by the bioinformatics predictions. Six new sites were revealed by the Ψ mapping. Three of them are guided by previously identified snoRNAs and one was matched to a newly identified snoRNA which is reported in this study for the first time. For the other two sites we failed to identify the H/ACA responsible for the modification. However, these Ψs must be guided by snoRNAs since we did not find these Ψs following *CBF5* silencing (Fig. 2a). These elusive snoRNAs may have a non-canonical or weaker binding sites and thus cannot be predicted using the stringent parameters that we were using for determining target snoRNA interactions. In addition, we failed to detect 7 Ψs on previously predicted positions (Supplementary Table S1). Two of these positions (SSU-Ψ1596, LSU3-Ψ578) are also methylated and hence the reverse transcriptase falls off before these positions, but this termination is not sensitive to CMC. The other 6 positions may also be the result of methylation or represent incorrect/non-accurate computational predictions. Interestingly, a few snoRNAs, such as TB11Cs4H2 and TB9Cs1H1, have the potential to guide 2–3 different Ψs on rRNA. The presence of a single hairpin dual function snoRNA that can potentially guide pseudouridylation on two sites was recently described by us in *Leishmania*^17^. Indeed, 17 rRNA-modifying-snoRNAs are predicted to also guide modifications on non-coding RNAs (U snRNA and tRNAs) (Supplementary Table S1) and others may have targets on other ncRNAs such as snoRNAs and 7SL RNA (our unpublished data) and possibly also on mRNAs.

Next, we examined the difference in the magnitude of the modification on each position between the two life cycle stages in and the three independent biological replicates. The results revealed that the level of an 8 Ψs was slightly reduced, but that the level of 21 Ψs was elevated between 1.3- to 2.7-fold in BSF (Supplementary Table S1). To appreciate the changes in pseudouridylation between the two stages we plotted the Ψ-fc fold change for those positions which are hypermodified in BSF in an arithmetic scale (Fig. 2b). Interestingly, in most of the cases more than a single modification was changed in the same domain and adjacent modifications were often elevated. Most significant were the four adjacent Ψs in H69 and four neighboring Ψs in H89, as well three positions in H90 and H92 located in the PTC.

### The small RNome of *T. brucei* identifies 83 H/ACA snoRNAs

**Figure 3:**
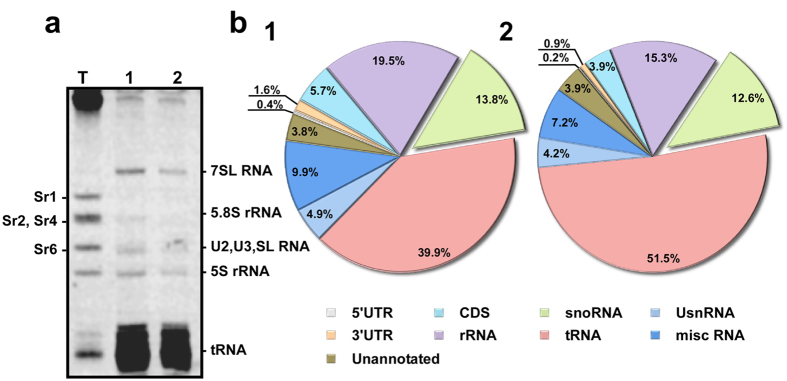
The small RNome libraries of BSF and PCF. **(a)** The pattern of the small RNAs present in post-ribosomal supernatant (PRS). Whole cell extracts from 10^9^ PCF and BSF cells were prepared and depleted from ribosomes and described in “Methods” section. The RNA was extracted from the PRS and separated on a 6% denaturing gel and stained with ethidium bromide. T. Total RNA, 1. PCF and 2. BSF RNA from post-ribosomal supernatant. **(b**) Pie diagrams describing the RNA content of the small RNA libraries. Annotation of the reads obtained from RNA-seq of the small RNome PCF and BSF libraries. The percentage of the different RNA molecules among the reads are summarized in the pie chart. The percentage reported is the mean of the two replicates for each RNA class. (1) PCF library; (2) BSF library.

To complete the snoRNA repertoire, as well as to explore if snoRNAs are developmentally regulated, we determined the small RNome of the procyclic and bloodstream forms. We reasoned that an elevated expression of the guide RNAs could explain the hypermodification pattern on rRNA in BSF. To this end, we prepared whole cell extracts from the two life stages, extracted the RNA from the post-ribosomal supernatant, and separated the small RNA fraction by electrophoresis on a denaturing polyacrylamide gel. The results (Fig. 3a) revealed the enrichment of distinct small RNAs such as of 7SL RNA^23^, tRNAs and small RNAs in the size of U2, U3 and SL RNA^24^. The snoRNAs cannot be seen in the gel, because they are masked by tRNAs. The purified RNA was used to prepare small RNA libraries as previously described^16^. Two sets of libraries (biological replicates) were sequenced with Illumina HiSeq resulting in 70 and 63 (PCF1 and BSF1), and 250 and 240 (PCF2 and BSF2) million reads mapping to the genome. The data in (Fig 3b) report the mean of the replicates for the different RNAs families which highlights the enrichment of small RNAs, and especially of snoRNAs. These libraries were reproducible, since a good correlation was found between the Reads Per Kilobase of transcript per Million mapped reads (RPKM) of the different RNAs; for example a correlation of r = 0.5, (p< = 0.001) was found between the snoRNAs abundance in the two libraries.

**Figure 4:**
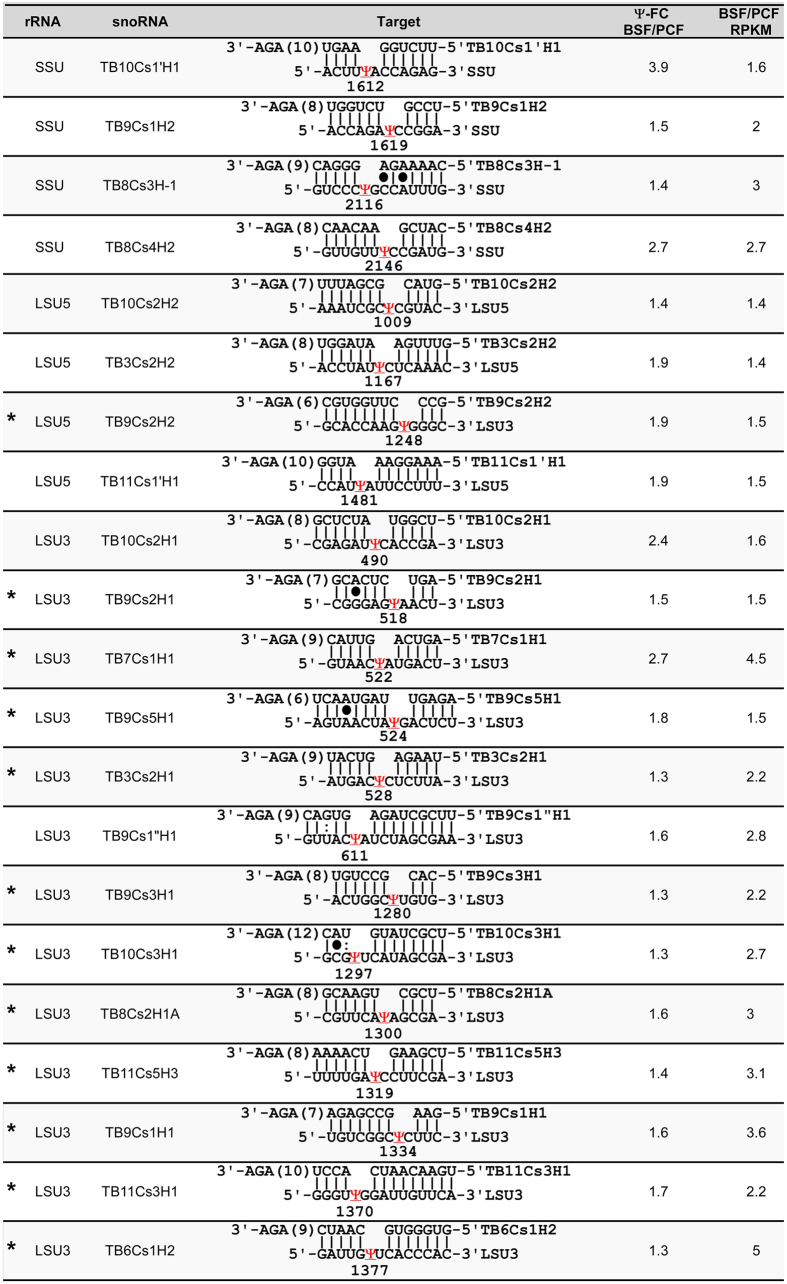
The identity of snoRNA target sites and differential expression of snoRNAs and the level of the guided hypermodified sites. The list contained information on the snoRNA guiding the hypermodified sites (more than 1.3 fold elevation in the BSF versus PCF). The identity and target of the snoRNAs are given. The ratio between the levels of Ψ in the two life stages is the average of three biological replicates of CMC libraries. For all snoRNAs, the differential expression of the BSF/PCF was calculated from the RPKM of each snoRNA in each PRS RNA-seq library based on the average of the two biological replicates. The Ψ-ratio is the Ψ-ratio of the treated samples (+CMC) divided by the Ψ-ratio in the non-treated samples (−CMC). The Ψ-ratio was computed for the PCF and BSF (mean of the three biological replicates for +CMC and –CMC treatment). The Ψ-ratio reported is the Ψ-ratio of the BSF versus PCF. The hypermodified Ψs with clear functional relevance are indicated with *.

The study identified 83 snoRNAs and their target (Supplementary Table S1). Next, we determined the differential expression of snoRNAs and the differential level of the Ψ they guide on rRNA in BSF and PCF. A list of snoRNAs which guide hypermodified positions in BSF by more than 1.3-fold compared to PCF (based on the two replicates) is given in (Fig. 4).

**Figure 5:**
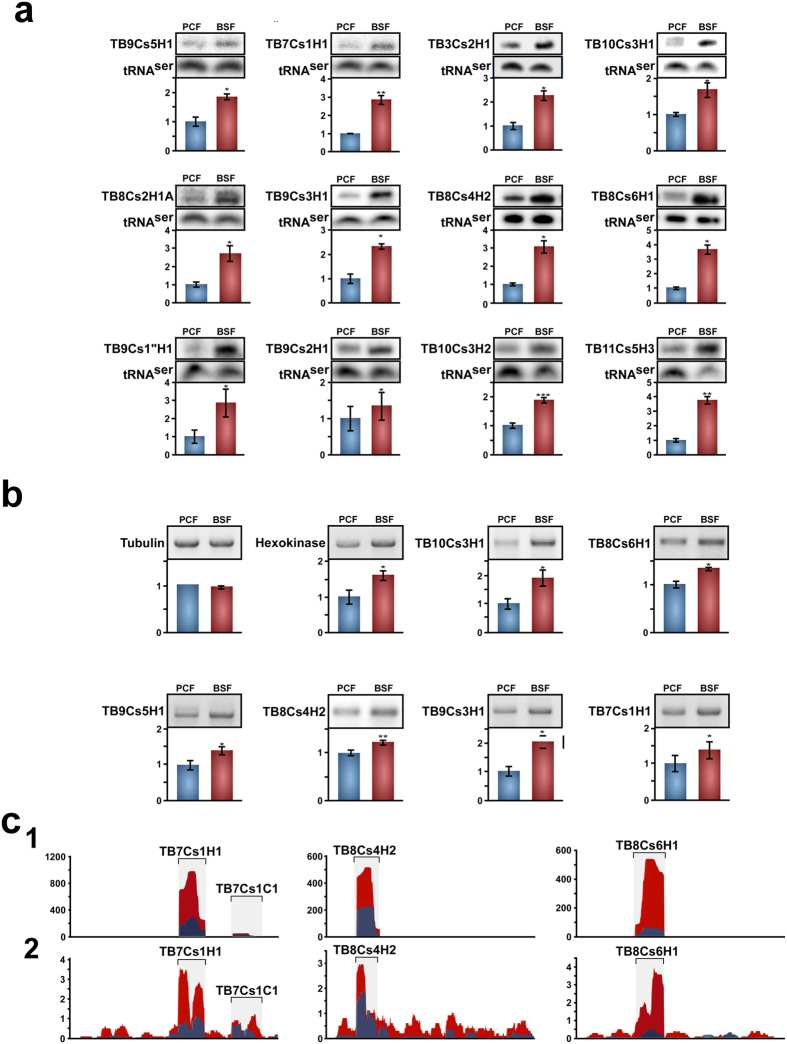
(**a**) Validation of elevation in snoRNA in BSF. Total RNA (10 μg) from both PCF and BSF was subjected to primer extension with primer specific to the snoRNA. The level of RNA was determined using the level of tRNA^Ser^ which was shown to be equally expressed in the two stages. The products were separated on a 6% denaturing gel. Data are represented as mean ± s.e.m. Experiments were done in triplicate (n = 3), *P < 0.05; **P < 0.01, ***P < 0.001 Student’s *t-*test. **(b)** Pre-snoRNA are elevated in BSF. RT-PCR was conducted as described in Methods using reverse primer complementary to the snoRNA and SL as the forward primer. Data are represented as mean ± s.e.m. Experiments were done in triplicate (n = 3), *P < 0.05; **P < 0.01. Student’s *t*-test. **(c)** Coverage of selected snoRNAs and their precursors. (1)- snoRNA coding. The read distribution profile of the snoRNA coding sequence based on the PCF1 (blue) and BSF1 (red) libraries. (2)- snoRNA precursor. The read distribution profile of the snoRNA precursor in PCF (blue) and BSF (red) is based on data obtained from^26^.

To verify the changes in the level of snoRNAs observed by RNA-seq of PCF and BSF libraries, the level of 12 snoRNAs which were found to be elevated in BSF was determined by primer extension and the statistically significant results are presented in (Fig. 5a). We utilized tRNA^Ser^ as a control for RNA amounts. Note that the tRNA level does not change between the two stages. Indeed our primer extension results supported the RNA-seq data demonstrating that the levels of these selected snoRNAs are elevated in the BSF.

In trypanosomes many of the snoRNA genes are organized in clusters carrying C/D and H/ACA genes. Specifically, ten snoRNA genes are present in 5 clusters containing only H/ACA snoRNA, and 30 are solitary and are located in genes encoding for a single H/ACA RNA^16^, enabling control of the H/ACA level without changing the C/D levels. Out of the 40 described H/ACA snoRNAs, 23 were shown to be up-regulated in BSF. snoRNAs present in clusters, as well as solitary snoRNAs, are processed from *trans-*spliced and polyadenylated transcripts by a still unknown mechanism^25^. One possibility, is that in BSF the level of these snoRNAs are elevated due to increase in the level of pre-snoRNA. To determine whether this is the case, we examined the levels of snoRNA precursors by RT-PCR for snoRNAs with up-regulated levels in BSF. The statistically significant results revealed an elevation in the level of pre-snoRNA in BSF (Fig. 5b). As a control, we examined the mRNA levels of tubulin and hexokinase. As expected, there was no change in the level of tubulin mRNA and there was an increase in the level of hexokinase mRNA, which is known to be elevated in BSF (Fig. 5b). Next, and in order to examine whether this up-regulation can be observed on a genome-wide scale, the level of the 30 solitary pre-snoRNAs was inspected using the previously published data of the transcriptome in the two life cycle stages^26^. However, spliced leader (SL) and poly (A) sites were only available for 13 solitary snoRNAs^25^. Indeed, the level of 12 of these pre-snoRNAs was elevated in BSF (two such examples are presented in (Fig. 5c2) which also resulted in an increase in the level of mature snoRNA (Fig. 5c1).

Since transcription regulation is scarce in trypanosomes^25^, up-regulation of the precursors may reflect regulation at the splicing and/or stability levels, which depends on flanking sequences to the RNA coding region. To search for motifs that might govern this regulation, we utilized the domain finding program MEME^27^. Briefly, we looked for overrepresented motifs in the sequences flanking the solitary up-regulated snoRNAs. Interestingly, we found a motif enriched in the 3′ flanking domain which is U-rich and highly similar to the PTB1 binding site^28^. However, PTB1 was not shown to be developmentally regulated and thus other RNA binding proteins that bind these sequences might participate in this regulation.

### The Ψ is enriched mainly in LSU 3′ domain H69, H89, H90 and H92

To gain insights into the possible effect of the increased Ψs on rRNA, the Ψs were positioned on the secondary structure of rRNA (Supplementary Fig. S1). The results indicated the presence of 40 Ψs on rRNA, which are also found in other eukaryotes, as well as trypanosome-specific modifications (31 Ψs) of which 17 are shared with *Leishmania major,* suggesting the presence of species-specific modifications (Supplementary Fig. S1).

**Figure 6:**
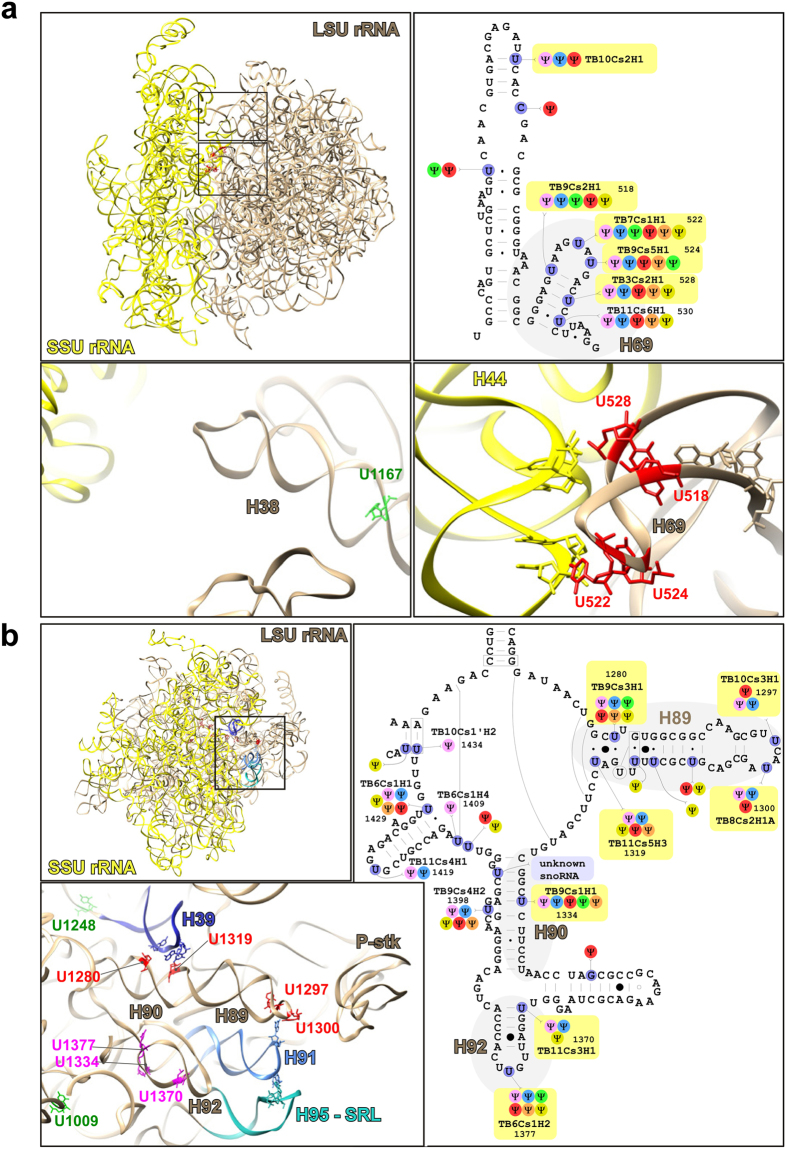
The location on the secondary structure and modeling of the hypermodified Ψ site s. (**a**) The location of hypermodified Ψs on H69 of LSU. The left panel illustrates the location of H69 and H38 in the atomic resolution rRNA structure^29^, (the SSU structure is in yellow and the LSU in brown). A magnified image of the H69 and H38 structures and the potential interactions of H69 with SSU H44 is illustrated below. The hypermodified positions on H69 are depicted in red and those on H38 in green. The right upper panel illustrates the secondary structure of H69. The hypermodified positions are circled in yellow; the snoRNA guiding the modifications are indicated. The modifications present in other eukaryotes are depicted in different colors; *T. brucei*, pink; *L. major*, blue; human, red; *Arabidopsis thaliana*, green; *S. cerevisiae*, brown; *Euglena gracilis*, yellow. (**b**) The location of hypermodified Ψs on LSU. The left panel illustrates the location of H89, H90, H92 of the PTC in the atomic resolution rRNA structure^29^ including a magnification of the structure and the potential interactions with H91 and H95 (SRL). The hypermodified positions of H89 are depicted in red, the hypermodified positions of H90 and H92 are depicted in purple and the hypermodified positions on 5′ LSU (H39) are depicted in green. The right panel illustrates the secondary structure of PTC. The hypermodified positions are circled in yellow; the snoRNAs guiding the modifications are indicated with colors as indicated in Panel a.

Of special interest is the finding that out of the 21 hypermodified positions, 8 were clustered in two domains. Four adjacent positions in H69 were shown to be elevated in BSF. In addition, four positions were shown to be hypermodified in H89. The structure of these domains is shown in (Fig. 6)^29^. In addition, the structure of the domains carrying hypermodified positions U1334, U1370, and U1377 present in H90 and H92 of PTC and that of U1167 of LSU 5′ are also presented (Fig. 6). For the possible significance of these hypermodifications for rRNA function see Discussion.

### Overexpression of four snoRNAs directing modification on H69 accelerate growth of PCF parasites at an elevated temperature

**Figure 7:**
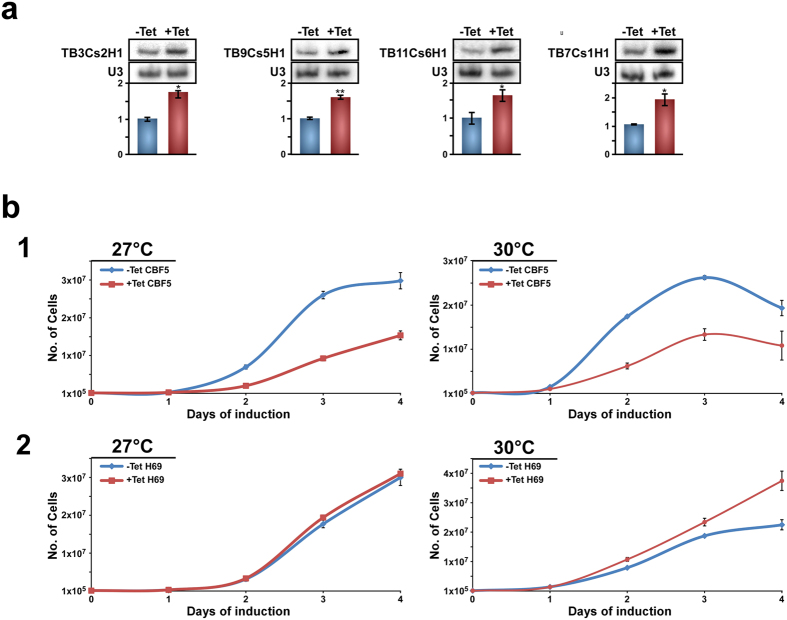
The effect on growth on parasites at different temperatures carrying the *CBF5* silencing construct and cells overexpressing the snoRNAs guiding modification on H69. (**a**). Northern analysis demonstrating the overexpression of the snoRNAs. Cells carrying the vector expressing the four snoRNA genes were induced for four days. RNA was subjected to Northern analysis with the indicated probes. Data are represented as mean ± s.e.m. Experiments were done in triplicate (n = 3), Data are represented as mean ± s.e.m. Experiments were done in triplicate (n = 3), *P < 0.05; **P < 0.01 Student’s *t-*test (**b**) (1) Growth of *CBF5* silenced cells. Uninduced cells carrying the *CBF5* silencing construct (-Tet) were compared with cells induced for silencing at 27 °C and 30 °C. The number of cells in the uninduced culture are plotted in blue and those of induced cultures in red. (2) Growth of cells overexpressing the snoRNAs guiding Ψs on H69. The cells carrying the construct of snoRNAs guiding modification on H69. The designation of the uninduced and induced cells are as in (1). Data are represented as mean ± s.e.m. Experiments were done in triplicate (n = 3).

The hypermodification in BSF may help the parasite to cope with the higher temperature faced in the mammalian host. To assess the ability of particular pseudouridines to orchestrate this regulation we decided to focus on distinct pseudouridylated positions on H69, which were found to affect translation efficiency, fidelity and even rRNA processing^13^. To this end, we synthesized a synthetic gene which contains the four snoRNAs (TB7Cs1H1, TB9Cs5H1, TB3Cs2H1, TB11Cs6H1) including their flanking intergenic regions (to assure their efficient and correct processing). The synthetic gene was cloned into the expression vector pLew100. The expression of the genes was induced by tetracycline addition. Overexpression of the snoRNAs was observed upon induction (Fig. 7). Next, the growth rate of the transgenic cells expressing the four snoRNAs before and after induction was monitored alongside cells silenced for *CBF5*. The experiment compared the growth rate at 27 °C to 30 °C. The results indicate that the growth of the cells in which *CBF5* has been silenced is more severely inhibited at 30 °C compared to 27 °C, suggesting that snoRNA function is essential for coping with growth at elevated temperature. Most relevant to this study is the finding that overexpression of the four snoRNAs guiding the modification on H69 slightly increased the growth rate of the parasites at 30 °C compared to uninduced cells, suggesting a role for the hypermodifications on H69 for growth at elevated temperatures (Fig. 7).

## Discussion

The recent studies and especially the Ψ-seq technology focused the attention on the importance of this modification for controlling gene expression^2^,^3^,^4^. However, our study is the first to indicate that pseudouridylation is developmentally regulated in two life cycle stages of *T. brucei* representing the insect vector and the mammalian host. The finding of very distinct changes in the level of Ψs in rRNA is intriguing, and this is the first study to suggest that rRNA pseudouridylation is not static, but is a regulated process. Our study further suggests the importance of particular positions possibly for ribosome function in different environments. The study demonstrates that overexpression of four snoRNA which guide modification on H69 and which are hypermodified in BSF accelerate the growth of PCF at an elevated temperature suggesting that hyper-pseudouridylation of rRNA in these critical and important positions contributes to the ability of the parasite to maintain ribosome function while cycling between the two hosts.

Trypanosome rRNA is hypermodified as compared to yeast with a similar genome size. We have previously suggested that the numerous 2′-*O-*methylations (Nms) in trypanosomes might stabilize the ribosome while cycling between its two hosts. Our hypothesis was based on the fact that the thermophilic *Archaea* displays extremely high levels of this modification, as well as on the finding that plants that are exposed to a gradient of temperature also possess many more Nms than metazoans with the same genome size^20^. In this study, we showed an increase in the level of H/ACA and a very distinct hyper-pseudouridylation on 21 positions on the rRNA in BSF. The hypermodified sites suggest that they play an important role in ribosome function in BSF. Indeed, this was demonstrated in an experiment where four of the snoRNA guiding hyper-pseudouridylation in BSF was shown to provide a slight advantage for growth of PCF parasites at 30 °C (Fig. 7).

Moreover, the elevation in hyper-pseudouridylation in the LSU rRNA of BSF occurs mainly at two regions involved in important ribosomal catalytic activities with the first region (H69) being close to the decoding center, and the second (H39, H89, H90 and H92) being close to the sarcin-ricin loop (SRL). The hyper-pseudouridylations in H69 take place at the stem-loop interacting with SSU H44 just below the A-site (Fig. 6a), thus forming an important ribosomal inter-subunit bridge (bridge 2a). H69 is also known to be involved in ribosome recycling^30^,^31^. Hyper-pseudouridylations occur on H39 (one U), helix H89 (four Us), H90 (one U) and H92 (two Us). Theses hyper-pseudouridylations could in theory induce conformational changes in H39 and H89, which could be propagated to the SRL, mediated by the interaction with H91 (Fig. 6b). In addition, the hypermodified residues on H90 and H92 of LSU 3′-end can also influence directly the conformation of H91 and therefore impact the SRL (Fig. 6b). The SRL is known to be implicated in the ribosome GTPase activity, involved in nearly all steps in translation regulation^32^. Another essential position is hypermodified U1167, which is located on the A-site finger (H38), forming one of the transient intersubunit bridges during the ribosome rotation and the SSU head swiveling, and hence may have an impact on the translation efficiency/rate (Fig. 6a). Some other hypermodifications, such as U1009 (Fig. 6b), are also located near the SRL region, however the connection with the latter is unclear.

The function of individual Ψs has been extensively studied in yeast ribosomes, either by affecting the process globally or by depleting individual modifications. As an example, deleting 5 H/ACA that guide modification in the PTC resulted in defects in protein synthesis and growth^12^. Seven of the modifications that were elevated in BSF are present in the PTC and four of the positions are conserved in other eukaryotes (Fig. 6). Interestingly, the two additional hypermodified positions located in H89 exist only in trypanosomatids and plants. The other domain that is hypermodified is in helix 69. This helix interacts with both A and P site tRNAs. Loss of three to five of these modifications in yeast caused the broadest defects observed for Ψs, such as reduced translation and its fidelity, and reduced rRNA level due to faster turnover^13^. Thus, it may be expected that overexpression of the snoRNAs which guide these important modifications could have a beneficial effect on the growth of the PCF parasite at an elevated temperature (Fig. 7). It would be interesting also to manipulate the pseudouridylation of other BSF hypermodified sites. One such site is located at the tip of PTC and is guided by TB6Cs1H2. In yeast, this site is guided by snR10. snR10 depleted cells are temperature sensitive and when the modification on this site was blocked, formation of 80S ribosomes on mRNA was compromised and translation activity was impaired^12^.

For many years Ψ formation was considered a constitutive process, but recent studies revealed that pseudouridylation is tightly regulated. In yeast, during heat shock or starvation, snR81, which catalyzes pseudouridylation on rRNA, directs pseudouridylation of U2 snRNA at position 93. This induced Ψ has a negative impact on pre-mRNA processing^14^. Most recently, studies using Ψ-seq identified an increase in mRNA Ψs during nutrient deprivation in yeast and serum starvation in human cells. As many as 42% of the yeast ~260 Ψs were shown to be regulated and are not present in log–phase but only in post-diauxic growth^3^. Interestingly, most of the heat-shock induced Ψs in yeast result from the activity of Pus7p which migrates from the nucleus to the cytoplasm under heat-shock^2^. The effect of pseudouridylation on developmental regulation was noticed recently and Pus1p pseudouridylation on U6 snRNA was shown to be induced during filamentation growth of yeast^33^. In addition, using enriched CeU-seq, a selective labeling and pull-down method in human RNAs, led to the identification of 2,084 Ψ sites in almost 2,000 transcripts. Interestingly, under different stresses such as heat-shock and oxidative stress, different mRNAs were hypermodified; in heat-shock hypermodification was found on mRNAs in transport and localization related functions whereas in oxidative stress on chromatin related functions. Many of the mRNA Ψ sites are mediated by hPUS1^5^. However, our study is the first to suggest that rRNA pseudouridylation is not static and that changes in the level of pseudouridylation take place during the natural life cycle of an organism.

Forty three H/ACA snoRNAs were found to be elevated in BSF, but their corresponding modifications on rRNA were not found to be hypermodified in BSF (Supplementary Table S1). To understand this puzzling observation we need to note that many H/ACA snoRNAs have a dual function and can potentially guide modification on more than a single target^17^. Based on *in situ* hybridization, we noticed that these snoRNAs are localized to the nucleolus but also to a Cajal-like body located near the nucleolus and are therefore sno/scaRNAs (our unpublished data). Thus, most of the elevated snoRNAs in BSF may impact on pseudouridylation of substrates other than rRNA, such as U snRNAs, tRNA, snoRNAs and possibly even mRNAs. We are currently performing Ψ-seq on these substrates. Since the snoRNA elevated in BSF modify more than a single target, this may explain why the increase in the level of snoRNA did not always result in elevation of the modification on rRNA.

It was always puzzling why so many of the H/AC snoRNA genes in trypanosomes are not present in clusters as are the majority of C/D snoRNAs genes , but are found as solitary genes. In this study, we found that many of these snoRNAs are elevated in BSF. Thus, the need to elevate certain subset of Ψs is mediated by increasing the expression of these individual pre-snoRNA transcripts. The mechanism of up-regulation of pre-snoRNA and whether it is related to enrichment of binding sites for a pyrimidine rich domains in the 3′ flanking sequencing of these pre-snoRNA must be further investigated.

Here we provide evidence that hypermodifications may contribute to rRNA stability enabling the ribosomes to function while cycling between the insect and mammalian host experiencing temperature changes of 10 °C. Another intriguing possibility is that the hypermodifications may also change the affinity of ribosomes to mRNA and thus may enable the preferential translation of developmentally regulated mRNAs.

The potential need to regulate ribosome function in cycling between two hosts is characteristic to many important infectious agents, such as other trypanosomatids like *Leishmania* and *Trypanosoma cruzi,* as well as *Plasmodium* species. It will be of great interest to determine if this stage-regulated pseudouridylation on rRNA is conserved in other trypanosomatids, as well as in other parasites. We anticipate that this stage-specific hypermodification in trypanosomes is more widespread and may include other non-coding RNAs or even mRNAs.

## Methods

### RNA-seq of snoRNAs and quantification of snoRNAs

The procyclic form *T. brucei* strain 29–13 was grown in SDM-79 medium^34^ supplemented with 10% fetal calf serum. The bloodstream form *T. brucei* strain 427, was aerobically cultivated at 37 °C under 5% CO_2_ in HM1-9 medium^35^. Whole cell extracts was prepared from 10^9^ cells as described^36^ and after extraction with 0.3M KCl, the ribosomes were removed by centrifugation for 2 h at 33,000 rev./min in a Beckman 70.1Ti rotor (150,000 ×g). RNA extracted from the post-ribosomal supernatant (PRS) was used for library preparation essentially as described and sequenced by Illumina sequencing^16^,^17^. The reads from the PCF and BSF libraries were mapped to the *T. brucei* Genome (version 5) using smalt v0.7.5 (http://www. sanger.ac.uk/resources/software/smalt/) with the default parameters, allowing non-unique reads to be mapped randomly to their best match in the genome. Raw read counts for each snoRNA were obtained using Multicov from the Bedtools suite (v 2.17.0)^37^. For each snoRNA that appears multiple times in the genome, the counts for each genomic location were combined. Reads Per Kilobase of transcript per Million mapped reads (RPKM) was utilized as the quantification method to obtain a measure for the expression of each snoRNA. To verify the differential level of the snoRNAs in BSF and PCF, total RNA (10μg) was subjected to primer extension analysis as described^36^. The RT-PCR to detect the pre-snoRNA was performed as described^16^.

### Detection of Pseudouridylated Sites

RNA from PCF and BSF (20 μg) was treated with CMC (N-cyclohexyl-N′-β-(4-methylmorpholinium) ethylcarbodiimide p-tosylate) in buffer (0.17 M CMC in 50 mM bicine, pH 8.3, 4 mM EDTA, 7 M urea) at 37 °C for 20 min. To remove all the CMC groups except those linked to the Ψ, the CMC-treated RNA was subjected to alkali hydrolysis with Na_2_CO_3_ (50 mM, pH 10. 4) at 37 °C for 4h, as previously described^22^. The RNA was then fragmented to a size range of 50–150 nt, adaptor was ligated to the 3′ end and cDNA was prepared using reverse transcriptase. Then, an adaptor was ligated to the cDNA, and after amplification the samples were sequenced in Illumina machine in paired end mode. The reads were mapped to *T. brucei* rRNA using smalt v0.7.5 (default parameters). Only properly paired mates were retained. Each read pair was “virtually” extended to cover the area from the beginning of the first read to the end of its mate. For each base, the number of reads initializing at that location as well as the number of reads covering the position were calculated. A combination of Bedtools^37^ and in-house Perl scripts were used to calculate the Ψ-ratio and Ψ-fc (fold change)^2^. For each nucleotide, we computed the Ψ-ratio, dividing the number of reads covering that nucleotide by the number of nucleotides initiating at the following base (i.e. corresponding to the last position copied by the reverse transcriptase). This was repeated for (−CMC) and (+CMC) samples. The Ψ-fc was computed as the log2-fold change of the Ψ-ratios in treated versus the non-treated samples. Applying a threshold, as previously described^2^, the putative Ψ sites were identified with the following requirements: a Ψ-fc (fold change) of 3 or greater, a Ψ-ratio of > = 0.01, and with a minimum of 5 reads initiating at the site. We applied this threshold to each sample. We then merged the positions to generate a list of all positions passing in at least one sample. For each developmental stage (PCF and BSF), we calculated a mean Ψ-ratio and Ψ-fc. We considered all sites which passed the criteria in at least one life stage.

### Construction of cell line overexpressing snoRNAs

A synthetic gene coding for the snoRNAs (see Supplementary Table S2) was synthesized including the flanking sequences and cloned into the pLew100 vector^38^. Transgenic cell lines were selected as previously described^36^.

### Modeling of the hyper-pseudouridylations on the *T. brucei* rRNA

The hyper-pseudouridylated residues were mapped on the *T. brucei* rRNA based on the atomic model of its full ribosome structure^29^ using Chimera UCSF modeling and molecular viewing program^39^.

## Additional Information

**How to cite this article**: Chikne, V. *et al.* A pseudouridylation switch in rRNA is implicated in ribosome function during the life cycle of *Trypanasoma brucei. Sci. Rep.* **6**, 25296; doi: 10.1038/srep25296 (2016).

## Supplementary Material

Supplementary Information

Supplementary Table S1

Supplementary Table S2
