# Supplementary material for: A pseudouridylation switch in rRNA is implicated in ribosome function during the life cycle of Trypanosoma brucei
Source: Sci Rep. 2016 May 4;6:25296. doi: 10.1038/srep25296 (PMC4855143; doi:10.1038/srep25296)
Supplement: Supplementary Information [file srep25296-s1.pdf]

A pseudouridylation switch in rRNA is implicated in ribosome function during the life cycle of Trypanosoma brucei

Vaibhav Chikne, Tirza Doniger, K. Shanmugha Rajan, Osnat Bartok, Dror Eliaz, Smadar Cohen Chalamish, Christian Tschudi, Ron Unger, Yaser Hashem, Sebastian Kadener, and Shulamit Michaeli.

Corresponding Author

Prof. Shulamit Michaeli

E-mail: shulamit.michaeli@biu.ac.il

Supplementary Information:

Supplementary Figure S1: Location of the pseudouridines on the rRNA .**a.** SSU, **b.** LSU 5' end, **c.** LSU3' end. The secondary structure was predicted based on the structure presented at **http://www/icmb.utexas/edu**. The designation of the colors are indicated in each panel.

Supplementary Table S1: The snoRNAs are listed based on the positions these target on rRNA. The base-pair interaction forming the pseudouridylation pocket on rRNA is presented. The RPKM for the snoRNAs of the different libraries are given. PC1 and PC2 are the two small RNA libraries prepared from the procyclic form (PCF) and BSF1, BSF2 are the two small RNA libraries prepared from the bloodstream form. The differential expression of the snoRNAs between the two life stages is given as ratio between the RPKM of BSF versus PCF. The extent of pseudouridylation guided by the snoRNA in the two stages was calculated by the ratio of Ψ-fc in BSF versus PCF as described in Fig. 2c. The additional site which a particular snoRNA may guide are indicated by the target and the position on the

target. The reference indicating where the snoRNA was first described is given. Several of the snoRNAs were not published before and their source (BS1 small RNA library described in this study) or small RNA library prepared from RNA selected by affinity purification with NHP2 (TbNHP2) are also indicated. * no significant pseudouridine detected; ** no significant pseudouridine detected, but methylation has been reported for the site; snoRNAs denoted with superscript numbers indicate they have multiple rRNA targets.[1-3]

Supplementary Table S2: A list of the oligonucleotides used for primer extension and RTPCR, construct and libraries preparation and Northern analysis.

References:

1 Myslyuk, I. *et al.* Psiscan: a computational approach to identify H/ACA-like and AGA-like non-coding RNA in trypanosomatid genomes. *BMC Bioinformatics* **9**, 471, doi:10.1186/1471-2105-9-471 (2008).

2 Liang, X. H. *et al.* A genome-wide analysis of C/D and H/ACA-like small nucleolar RNAs in Trypanosoma brucei reveals a trypanosome-specific pattern of rRNA modification. *RNA* **11**, 619-645, doi:10.1261/rna.7174805 (2005).

3 Doniger, T., Michaeli, S. & Unger, R. Families of H/ACA ncRNA molecules in trypanosomatids. *RNA Biol* **6**, 370-374 (2009).

Supplementary Figure S1

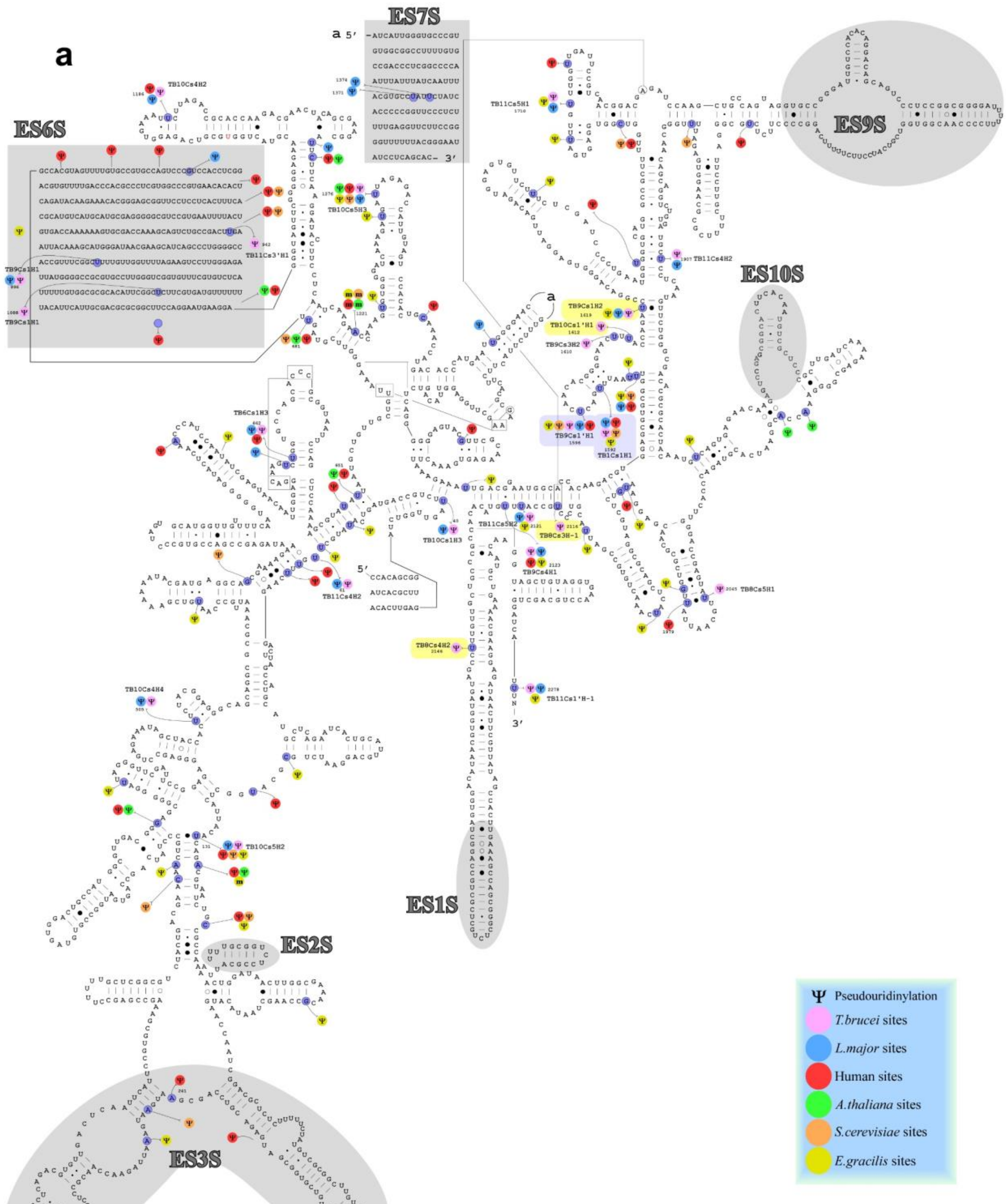

Trypanosoma brucei rRNA SSU

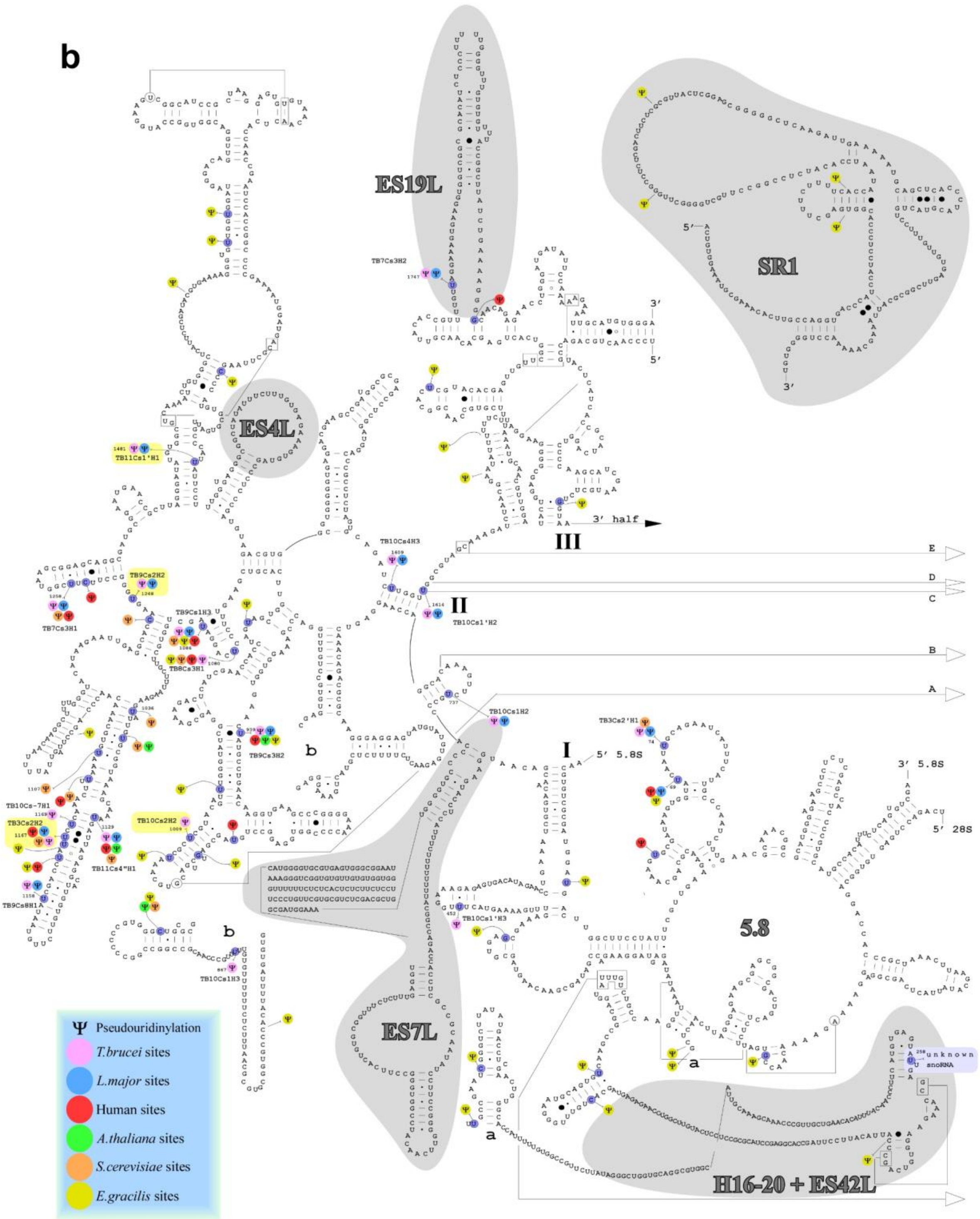

Trypanosoma brucei rRNA LSU5' half

c

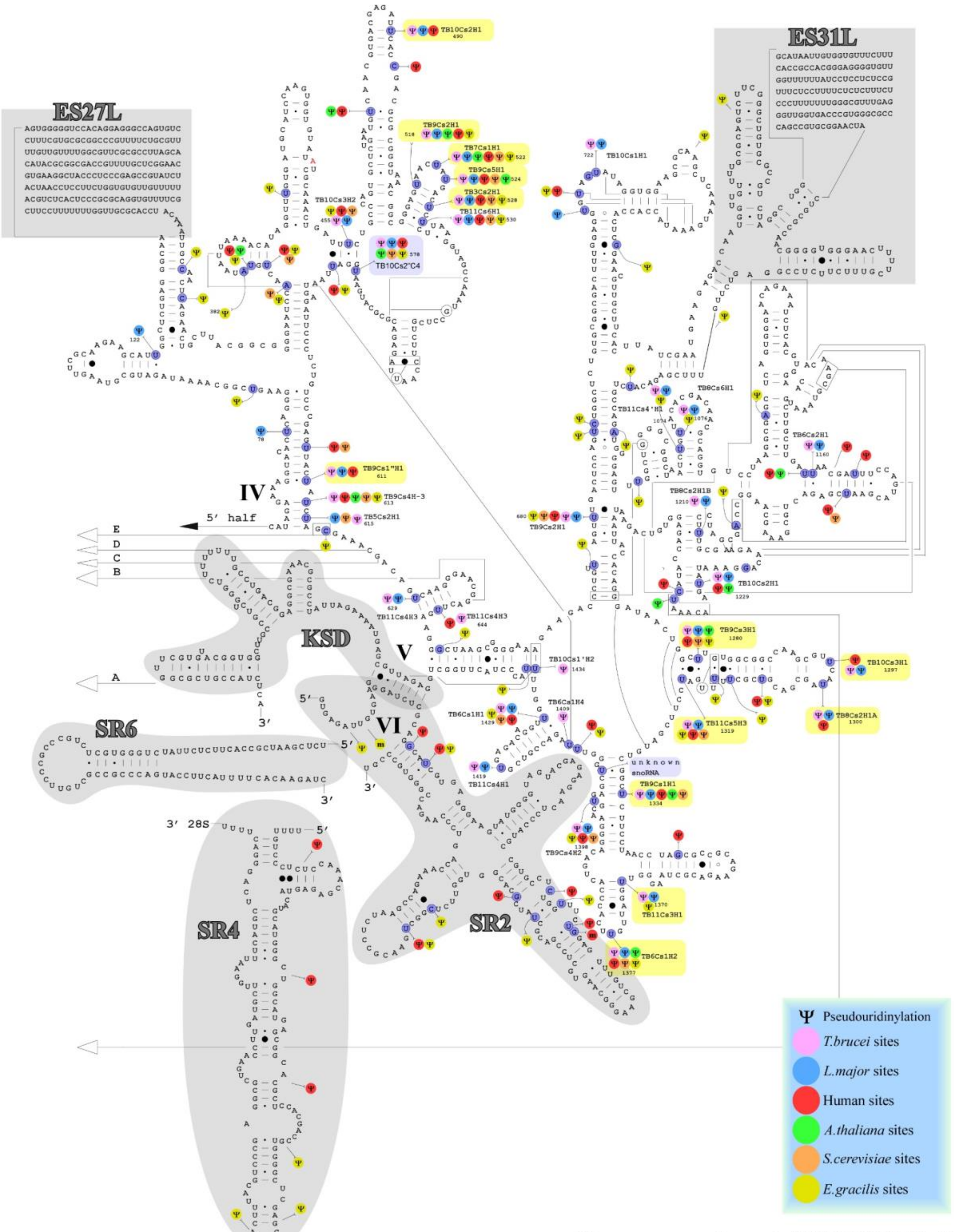

Trypanosoma brucei rRNA LSU3' half
